# The Ethics of Teaching: Critical Thinking and Reflection to Promote Professionalism by Mitigating Biases Including those against other Healthcare Professions

**DOI:** 10.3389/fphar.2016.00056

**Published:** 2016-03-14

**Authors:** Lon J. Van Winkle

**Affiliations:** Department of Biochemistry, Midwestern UniversityDowners Grove, IL, USA

**Keywords:** professionalism, inter-professional collaboration, critical thinking, critical reflection, mitigating biases, healthcare professions students

In the USA I see bumper stickers and t-shirts that declare “Critical thinking: the other national deficit.” Unfortunately here, and elsewhere in the world, these words are often true. Not only does the internet give us ample opportunity to express ourselves without much reflection or thought, but college and university courses do not always expect and foster critical thinking and reflection on the part of students, particularly when the courses have lecture formats and large enrollments. In my view, this is especially true of the basic and even the clinical sciences in healthcare professional education. Many of the latter educators do not heed well the admonition in McKeachie's Teaching Tips (McKeachie and Svinicki, 2014) “Teach your students to reflect both in and out of class. That reflection should never stop, because conscious reflection on values is perhaps the cornerstone of the ethics of teaching.”

But why is such thought and reflection important in education? Major struggles for all people are the biases and judgments they hold against others especially those outside their own personal groups. For example, in the New York Times a couple of years ago, Kristof (2014) wrote in his editorial “Some readers collectively hissed after I wrote a week ago about the need for early-childhood interventions to broaden opportunity in America. I focused on a 3-year-old boy in West Virginia named Johnny Weethee whose hearing impairment had gone undetected, leading him to suffer speech and development problems that may dog him for the rest of his life. A photo of Johnny and his mom, Truffles Weethee, accompanied the column and readers honed in on Truffles' tattoos and weight (instead of possible opportunities to help Johnny and other such children)…Why didn't readers see a caring mom instead of her…tattoos?”

We believe that such biases can be mitigated through exposure of students in our courses to people outside their groups especially when the students then write critical reflections about their experiences. Our evidence, from surveys of healthcare professional students (as well as from using formally validated instruments as discussed below), indicates that such activities by students in interdisciplinary teams seem to mitigate biases not only against patients but also against other healthcare professions. For example, 76% of medical students agreed whereas only 10% disagreed with the survey statement “Encounters with people in our team community service project helped me to see my potential biases toward patients more clearly regardless of the setting.” Similarly, 77% of pharmacy students and 66% of prospective health-care professions students agreed with the statement. An extremely important aspect of these service projects included regular and written critical thinking and reflection by students about their projects throughout the term in which the projects were performed.

We have shown elsewhere that such thought and reflection also foster patient-centered beliefs among students and even student-centered beliefs in faculty members (Van Winkle et al., 2011a,b,c). Similarly, critical thinking and reflection about elderly patients (Van Winkle et al., 2012a) and patients in minority groups (Van Winkle et al., 2013a) improved healthcare professional students' empathy scores. Empathy scores also improved among healthcare professional students performing critical thinking and reflection on their team service learning projects (Van Winkle et al., 2013b, 2014).

More important to the present theme, this critical thinking and reflection also likely mitigate biases against other healthcare professions students, when the thinking and reflection are performed as members of interdisciplinary healthcare professional teams. In a single 50-min biochemistry workshop, teams of medical and pharmacy students shared their critical thoughts and reflections on the roles of the other profession in the care of two patients (Van Winkle et al., 2012b). Completion of the workshop was associated with an increase in the physician-pharmacist collaboration scores of both groups of healthcare professions students, although the effect on pharmacy students was greater than the influence on medical students. When the time working on interdisciplinary teams was extended, from a single workshop, to 18 workshops over two quarter-term biochemistry courses, the collaboration scores of prospective healthcare professional students (primarily prospective medical and dental students) increased much more dramatically and approached the already high scores of pharmacy students (Van Winkle et al., 2013b). More revealing are the written thoughts and reflections of students about their work on inter-professional teams. As shown in the samples below, these reflections often dealt with mitigation of biases against the other profession. Nearly full reflections are included to give the reader the full depth of the sample students' experiences, thoughts and feelings. Such mitigation by most inter-professional team members likely contributed to the improved collaboration scores of all groups of healthcare professional students (Van Winkle et al., 2012b, 2013b).

For example, a pharmacy student wrote that “in life it is easy to recognize when you are right, but admitting you are wrong can be a different story…Going into workshop I had a negative preconception about doctors and their role within the healthcare team. I have spent years working in a pharmacy and I have become familiar with the drama that follows the doctors…The arrogance that comes with that position is often times too much to stomach…Every time a call had to be put into the doctor's office from the pharmacy it (was) like summoning the king/queen for a meeting with the local pauper. Returned calls often were short and degrading for something as simple as a forgotten signature or number of refills. With that said I felt as though I was in for a long 50 min meeting with the future doctors from Midwestern…When we read the Henrietta Lacks book I was not surprised with the way the doctors treated Henrietta because I felt that coincided with my ideas of doctors I have dealt with. However, a change was about to take place that altered my view of doctors for the better.

The Cameron Lord video really struck me because for the first time I saw a doctor act on the family's wishes rather than his own agenda. The doctor in the video took the time to find out what the best treatment options were based on the family's wants and made sure that decisions were made on their terms. In my opinion he seemed like a friend or neighbor first and a doctor second. Furthermore, the medical students from Midwestern understood my position, and surprisingly agreed with me about the current attitudes (of) doctors in the healthcare team. I was shocked when they used words like collaborate, incorporate, and teamwork when talking about the doctors of the future. While I initially felt (that) maybe they were putting on a show it hit me when one of the medical students said “on behalf of my profession I'm sorry.” It was at that moment I understood that I was the one who was being unfair to the doctors not the other way around.

Going forward, I feel that I can better live up to my values in future collaborations with other health care professionals by listening first and judging later. Too often I feel that I allow my previous misconceptions to determine how I treat others in the medical field. In order to better serve my patients I need to realize that the patient comes first and I come second. I need to create my feelings for others based on my individual interactions rather than the (previous) feelings I have created based on (prior) meetings with others. No two people are the same and while the saying goes “one bad apple spoils the bunch” in order to provide exceptional patient care I must understand that when I judge others I am the “bad apple.” Looking at the bigger picture of patient care and understanding that we are all working toward the same goals I can accept others on their own actions and not on the actions of those whom have come before.”

Similarly, an aspiring physician said “my first exposure to a faculty clinician left me with an impression. I quoted him as saying in my notes “everyone is very big on this team mentality toward healthcare, but be wary of this. The pharmacist, physical therapist, nurse, etc…will increasingly press for more responsibility and more input on decision making, but as soon as the lawsuit comes in, the buck stops with you, the physician.” This left me quit hardened. In the moment, I built a barrier when dealing with healthcare teams…to make sure that I would not be troubled by…the input of someone with training other than mine. I don't doubt that I am egotistical. I think all medical students are in a way, after all we are a select few that made it to this point but after talking with the pharmacy students, I think I need to work less on building barriers and more on giving our patients the superior care they deserve. This requires that we work with other professionals as a team.

The beginning of our conversation (with pharmacy students) was very cordial, after all, at this point we are all students and we talked about the rigors of our education. It was a rallying point that we all enjoyed, basically complaining about the number of tests, lectures, etc…to someone other than a student going through the same thing. It didn't take long however, for that early compatibility to fade. The pharmacy student I spoke with first was the person that changed me most…Initially I figured the conversation would go like this:

Pharmacy student: “I want to be included in more decision making and to not be treated like a subordinate.”

Medical student: “That's fine but are you willing to take the responsibility for say, the drug choice you recommend for me to prescribe to my patient?”

Pharmacy student: “Well in the end you prescribe so you should still have to take the repercussions.”

As you can see from the conversation I was expecting, I was cynical. What really happened was more like this:

Pharmacy student: “I want to be included on rounds in the hospital, I think my expertise on medications would be of great use to the medical team.”

Medical student: “Are you then willing to take the responsibility for the drug you recommended?”

Pharmacy student: “Absolutely, if the team makes the decision, the team should be responsible for the outcome. Besides that, if we work together, you concentrating on diagnosing, and deciding what the patient needs to get better, and I giving you the drug to complete the (treatment), we can then be more efficient in healing patients, and be more successful…The responsibility we share will be for…success, instead of failure.”

Medical student: “Wow…”

I was stunned, it was so far from what I was expecting. He was completely right…We share a goal, I want to be successful in treating patients as does he. I realized that my egotistical viewpoint, that everyone wants power but no responsibility was grossly unfounded. I realized that it's not fair to myself to place all of the responsibility on myself. It is also unethical to my patient, as giving them the best treatment, requires me to be more willing to work with other professionals like pharmacists. This certainly was eye-opening. With utmost respect, I think that even…seasoned clinicians should get a lesson like this.”

In the same spirit, I suggest the following modification of the quote above from McKeachie's Teaching Tips (McKeachie and Svinicki, 2014). This revision is intended for all healthcare professionals whether they are students or practitioners. The modification is: Learn to reflect alone and in teams when patients are present and when they are not. That reflection should never stop, because conscious and critical thought and reflection on values is perhaps the cornerstone of mitigating biases against patients and other healthcare professions.

## Author contributions

The author confirms being the sole contributor of this work and approved it for publication.

### Conflict of interest statement

The author declares that the research was conducted in the absence of any commercial or financial relationships that could be construed as a potential conflict of interest.

## References

[B1] Kristof N. (2014). The compassion gap. The New York Times. Sunday Review, March 2.

[B2] McKeachie W. J. Svinicki M. (2014). McKeachie's Teaching Tips. Belmont, CA: Wadsworth.

[B3] Van Winkle L. J. Bjork B. C. Chandar N. Cornell S. Fjortoft N. Green J. M.. (2012b). Interprofessional workshop to improve mutual understanding between pharmacy and medical students. Am. J. Pharm. Educ. 76:150. 10.5688/ajpe768150 23129849 PMC3475779

[B4] Van Winkle L. J. Burdick P. Bjork B. C. Chandar N. Green J. M. Lynch S. M. (2014). Critical thinking and reflection on community service for a medical biochemistry course raise students' empathy, patient-centered orientation, and examination scores. Med. Sci. Educator 24, 279–290. 10.1007/s40670-014-0049-7

[B5] Van Winkle L. J. Chandar N. Green J. M. Lynch S. M. Viselli S. M. Burdick P. (2011b). Does critical reflection by biochemistry learning teams foster patient-centered beliefs among medical students? Med. Sci. Educator 21, 158–168. 10.1007/BF03341613

[B6] Van Winkle L. J. Cornell S. Fjortoft N. Bjork B. C. Chandar N. Green J. M.. (2013b). Critical thinking and reflection exercises in a biochemistry course to improve prospective health professions students' attitudes toward physician-pharmacist collaboration. Am. J. Pharm. Educ. 77:169. 10.5688/ajpe778169 24159210 PMC3806953

[B7] Van Winkle L. J. Dobie S. Ross V. R. Sharma U. Green J. M. Lynch S. M. (2011c). Acute intervention to foster reflection on reciprocity in relationships increased participants' patient-or student-centered orientation scores in association with a medical biochemistry course. Internet. J. Med. Educ. 1 Available online at: http://ispub.com/IJME/1/2/7608

[B8] Van Winkle L. J. Fjortoft N. Hojat M. (2012a). Impact of a workshop about aging on the empathy scores of pharmacy and medical students. Am. J. Pharm. Educ. 76:9. 10.5688/ajpe7619 22412208 PMC3298407

[B9] Van Winkle L. J. La Salle S. Richardson L. Bjork B. C. Burdick P. Chandar N. (2013a). Challenging medical students to confront their biases: a case study simulation approach. Med. Sci. Educator 23, 217–224. 10.1007/BF03341624

[B10] Van Winkle L. J. Robson C. Chandar N. Green J. M. Viselli S. M. Donovan K. (2011a). Use of poems written by physicians to elicit critical reflection by students in a medical biochemistry course. J. Learn. Through Arts 7 Available online at: http://escholarship.org/uc/item/7513c5mv

